# Acupuncture needle fragments identified on X-ray and computed tomography studies of chest

**DOI:** 10.1590/0100-3984.2015.0142

**Published:** 2018

**Authors:** Lilian Fonseca Lima, Pablo Rydz Pinheiro Santana, Antonio Carlos Portugal Gomes

**Affiliations:** 1 Hospital Beneficência Portuguesa de São Paulo - Medimagem, São Paulo, SP, Brazil

**Figure 1:**
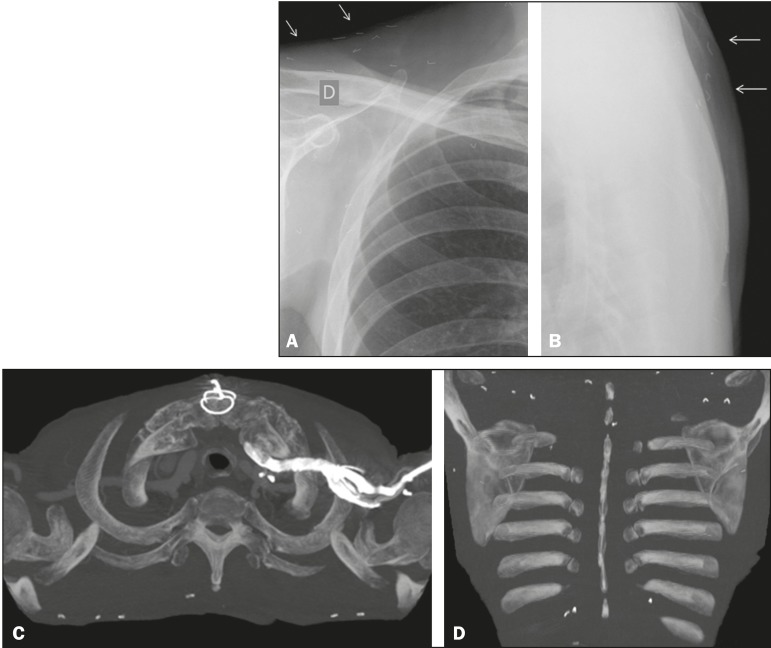
Chest X-rays, in frontal and profile views (**A** and **B**, respectively), showing small metallic objects in the subcutaneous tissue of the back and supraclavicular region, with similar sizes and varied forms, some being linear and others having some degree of curvature. **C,D:** Axial positron emission tomography/computed tomography of the thorax, showing small objects, the density of which is consistent with metal, in the subcutaneous tissue, predominantly in the back.

Dear Editor,

A 75-year-old male patient underwent chest X-ray and computed tomography of the thorax (Figure 1) for postoperative evaluation of myocardial revascularization. Small metallic objects were identified in the subcutaneous tissue of the back. The objects were similar in size but varied in form; some were linear and others had some degree of curvature. Those findings are consistent with acupuncture needle fragments.

Traditional Chinese acupuncture has been practiced for millennia. Approximately 40 years ago, it was introduced into medical practice in Brazil, where it is now widely used for the prevention and treatment of chronic pain. It consists in the insertion of needles into the subcutaneous tissue; the needles are left in place for up to 15 min, after which they are removed^1^. In some acupuncture modalities, the needles are inserted into the subcutaneous tissue and the protruding part of each is cut off; the remaining fragments are thus permanently maintained in the tissue, providing continuous neurological stimulation^2^. The needles have a maximum diameter of approximately 1 mm and a maximum length of 1.5 cm^3^. The needles are preferably made of gold, although they can be silver or stainless steel. The number of fragments varies and can be in the thousands.

In general, acupuncture needle fragments do not cause complications and appear as incidental findings on imaging examinations. They present as small, straight, curvilinear, or semicircular objects, of similar sizes, and can be confused with metallic sutures or clips. Occasionally, these structures can form foreign body granulomas and can even migrate, especially in patients without much subcutaneous fat^4^.

Although rare, complications resulting from traditional Chinese acupuncture have been the subject of two systematic reviews^5,6^. When such complications are severe, they are usually attributable to improper manipulation at sites where there is a high risk of injury to the adjacent organs and structures, which can result in pneumothorax, cardiac tamponade, or spinal injury^5^. They can also be related to the incidental breaking of a needle, which requires surgical removal in some cases^6^.

A review of the literature on acupuncture needle fragments remaining in the body of patients identified 29 articles on the topic. Those articles describe fragments that have migrated to numerous sites, such as the urinary bladder, shoulder girdle, spinal cord, right ventricle, L5 nerve root, bulb, carpal tunnel, liver, pancreas, stomach, colon, lungs, and kidneys^7^. In cases in which the patients underwent surgery for the removal of the fragments, there were no major postoperative complications. Acupuncture has also been shown to increase bone activity on scintigraphy.

The true prevalence of acupuncture needle fragments remaining in the body is unknown. It is possible that the condition is underdiagnosed because many affected individuals never undergo imaging examinations of the areas treated. Likewise, the prevalence of complications associated with acupuncture remains unknown. To date, there have been few studies of this specific topic. When acupuncture needle fragments appear as incidental findings on imaging examinations, they are regarded as a medical curiosity. Therefore, knowledge of their imaging aspects can be quite useful for radiologists.
